# Sleep Disruption and Atrial Fibrillation: Evidence, Mechanisms and Clinical Implications

**DOI:** 10.1161/CIRCRESAHA.125.325612

**Published:** 2025-08-15

**Authors:** Abhishek Deshmukh, Naima Covassin, Yves Dauvilliers, Virend K. Somers

**Affiliations:** Department of Cardiovascular Medicine, Mayo Clinic, Rochester, MN (A.D., N.C., V.K.S.).; Department of Neurology, Sleep-Wake Disorders Center, Gui-de-Chauliac Hospital, Centre Hospitalier Universitaire (CHU) Montpellier, Institute for Neurosciences of Montpellier Institut National de la Santé et de la Recherche Médicale (INSERM), University of Montpellier, France (Y.D.).

**Keywords:** atrial fibrillation, continuous positive airway pressure, heart failure, sleep apnea, central, sleep apnea, obstructive

## Abstract

Atrial fibrillation (AF) is the most prevalent sustained cardiac arrhythmia, with its incidence rising due to aging populations, obesity, and advancements in diagnostic modalities. The interplay between sleep disorders and AF is increasingly recognized, with obstructive sleep apnea (OSA) serving as a well-established risk factor. However, emerging evidence implicates additional sleep disturbances—including central sleep apnea, insomnia, and restless legs syndrome—in AF pathogenesis and progression. Despite compelling observational data, interventional studies evaluating the impact of sleep disorder treatment on AF outcomes have yielded mixed results. Although continuous positive airway pressure therapy in patients with OSA mitigates AF recurrence, randomized controlled trials have yet to confirm a definitive causal benefit. This review synthesizes epidemiological, mechanistic, and interventional data linking sleep disorders to AF.

Atrial fibrillation (AF) is the most frequent arrhythmia in clinical practice. With an aging population, growing rates of obesity, and better diagnostics, including wearables and implantable loop recorders, the anticipated number of patients seeking care for AF will continue to increase. There is an evolving interest in modulating the atrial substrate and triggers by optimum risk factor modification. The intersection of obstructive sleep apnea (OSA), obesity, hypertension, and AF is well-known. Patients with OSA are more likely to have AF, and more patients are getting formally screened and diagnosed with AF. However, other sleep disorders, including central sleep apnea (CSA), insomnia, sleep insufficiency, and restless legs syndrome (RLS), have also been implicated in the genesis of AF and AF-related sequelae. In this review, we present observational and experimental evidence linking sleep disruption and AF, discussing pathophysiology, treatment effects, and the overall impact of disrupted sleep in managing patients with AF. Given the high prevalence of both OSA and AF, their frequent comorbidity, and the extensive body of research investigating their interactions, the primary focus will be on the OSA-AF relationship.

## OSA and AF

### Epidemiology

Several epidemiological studies highlight the association between OSA and obesity, diabetes, hypertension, coronary artery disease, congestive heart failure (HF), stroke, and various arrhythmias.^1–3^ There is heterogeneity in the prevalence and incidence of OSA, as these depend on the metrics used to test for OSA. However, the absolute and causative common link between sleep apnea and AF is likely the patient’s overall cardiovascular health, which determines the substrate for AF.^4^

Over the past 50 years, the prevalence of AF has increased 3-fold.^5^ The Global Burden of Disease project estimated a worldwide prevalence of AF at around 46.3 million individuals in 2016.^6^ Three to 6 million Americans have AF, projected to reach between 6 and 16 million by 2050.^7^ The overall rates of hospitalizations, repeat hospitalizations, and healthcare utilization are increasing due to the duration of hospital stays and dollars spent.^8–10^

OSA is frequently seen in patients with AF.^11–13^ Patients diagnosed with OSA have a 2-to-4-fold higher risk of developing AF when compared with those without OSA. Similarly, when tested for OSA, individuals with AF have an increased prevalence ranging from 10% to 60%.^14–16^ A robust comparison of the causation and association of OSA and AF is nebulous due to nonuniform definitions of both AF and OSA and further confounded by variable screening and diagnostic tests. The prevalence of OSA (Apnea-Hypopnea Index [AHI] ≥5) is reported between 43% to 85% among patients undergoing diagnostic testing, whether at home or in a dedicated sleep laboratory ^17,18^

The Multi-Ethnic Study of the Atherosclerosis cohort demonstrated that AF was prevalent in severe OSA at ≈7.5%.^19^ In the Sleep Heart Health Study, patients with severe OSA (AHI≥30) had an age, sex, body mass index (BMI), and prevalent cardiovascular disease (CVD)–adjusted odds ratio of 4.0 (95% CI, 1.0–15.7) for the presence of AF compared with AHI<5.^20^ The Sleep Disorders in Older Men Study highlighted a dose-dependent association of OSA with prevalent AF.^21^

Beyond the associative or causative relationship, it is essential to note that the frequency of AF is higher after an obstructive breathing event. Paroxysmal AF events are more likely to occur during the 90 seconds after an obstructive event than during a time frame without an obstructive event (odds ratio, 17.9 [95% CI, 2.2–144.2]).^22^ The severity of the nightly obstructive burden also predicts changes in AF burden.^23^

## Mechanistic Link Between OSA and AF

**Figure 1.**
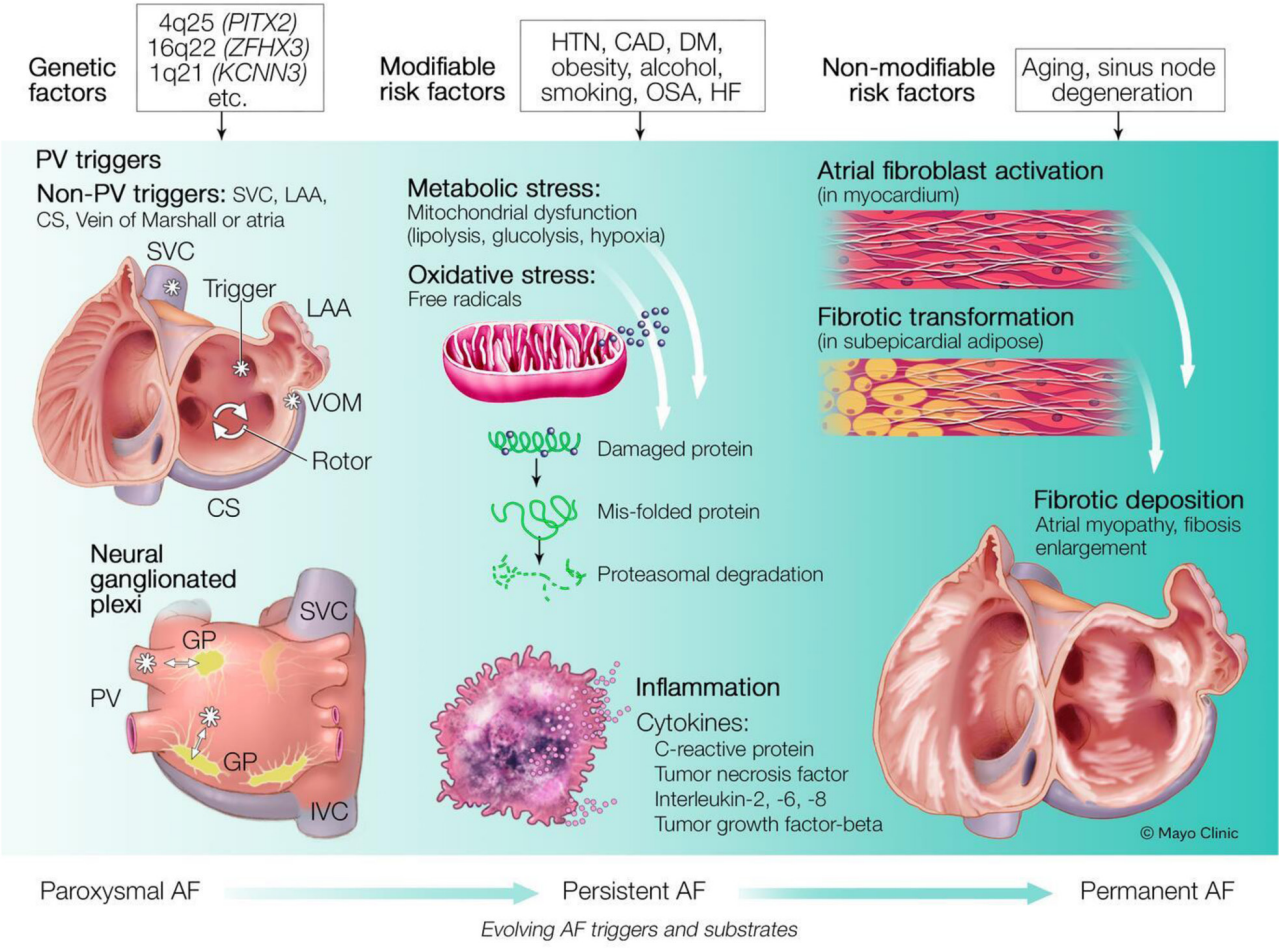
**Pathophysiology and progression of atrial fibrillation linking genetic, modifiable, and nonmodifiable risk factors.** AF indicates atrial fibrillation; CAD, coronary artery disease; CS, coronary sinus; DM, diabetes; GP, ganglionated plexi; HF, heart failure; HTN, hypertension; IVC, inferior vena cava; LAA, left atrial appendage; OSA, obstructive sleep apnea; PV, pulmonary vein; SVC, superior vena cava; and VOM, vein of Marshall.

**Figure 2.**
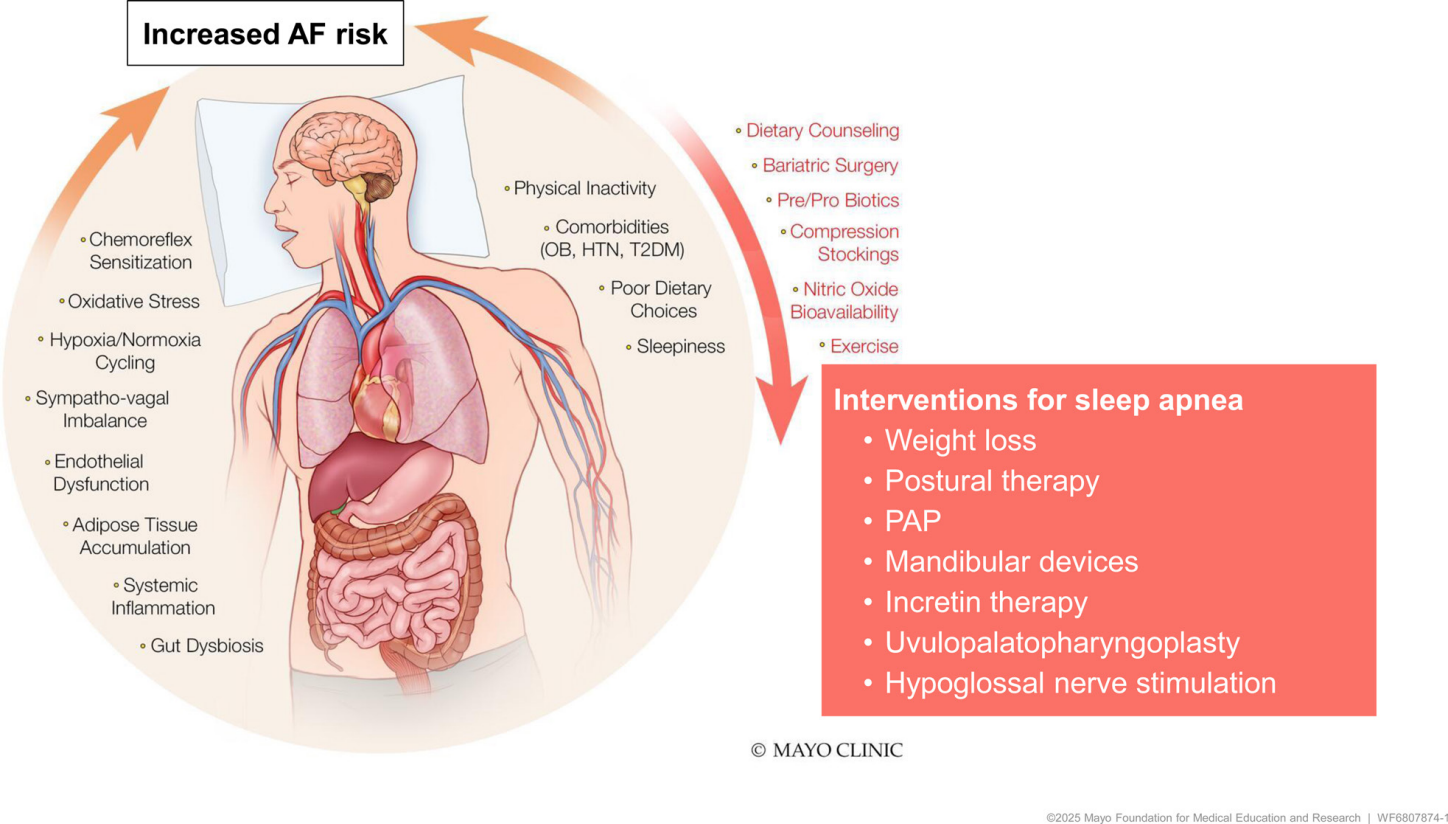
**Link between obstructive sleep apnea and increased risk for cardiovascular diseases and atrial fibrillation.** AF indicates atrial fibrillation; HTN, hypertension; OB, obesity; PAP, positive airway pressure; and T2DM, type 2 diabetes.

The elucidation of the exact mechanism(s) of AF is a work in progress. Moe and colleagues are credited with the original computer-based mathematical model of AF using the multiple-wavelet concept of AF.^24^ The current paradigm of the genesis of AF is based on Moe’s multiple reentrant wavelets and discharging automatic foci model. Current rhythm control approaches for treating AF include targeting cardiac ion channels with antiarrhythmic medications and targeting pulmonary and nonpulmonary vein triggers by catheter ablation. However, we still have not found the elusive cure for AF compared with other reentrant supraventricular arrhythmias.^25^ This begs the question of whether the treatment options are not ideal or whether the potential therapeutic targets have yet to be identified. Recent seminal work by Nalliah et al,^26^ Hendriks et al,^27^ and Chung et al^28^ demonstrates that the primary emphasis on risk factor modification has further improved clinical outcomes of AF by mitigating the progression of the atrial substrate (Figure 1) Specific mechanisms underlying the association between OSA and AF are outlined below and illustrated in Figure 2.

## Synergistic Effects of Obesity and OSA on AF Risk

Obesity and OSA frequently co-occur in patients with AF, and together they markedly amplify AF risk. Obesity itself is a strong risk factor for AF. The pathophysiological interplay between these conditions has garnered considerable attention due to the increasing prevalence of obesity and the robust evidence linking OSA with AF.^15^ The Framingham Study demonstrated that each unit increase in BMI increases the risk of AF by ≈4%.^29^ Emerging evidence suggests that OSA may mediate some of the AF risk conferred by obesity.^30^ Obesity mediates AF by structural and electrical remodeling, including adiposity, neurohormonal changes, inflammation, fibrosis, and oxidative stress. Key among these is the accumulation of epicardial adipose tissue. This tissue, through paracrine signaling and the release of adipokines, plays a crucial role in promoting inflammation and fibrosis within the atrial tissue. Epicardial adipose tissue volume, particularly around the left atrium, has been linked to increased fibrosis, dilatation, and the structural remodeling seen in AF. The role of epicardial adipose tissue in this process is pivotal, as it influences the electrical properties of the atrium by secreting proinflammatory cytokines and contributing to myocardial fibrosis. Moreover, obesity-induced inflammation activates the NLRP3 (NLR family pyrin domain–containing 3) inflammasome, which further drives fibrosis and arrhythmogenic substrate formation.^31^

The clinical implications of the intersection between OSA, obesity, and AF are profound. Obesity-related OSA is associated with a higher burden of arrhythmias, particularly in those with concomitant risk factors for AF, such as hypertension, diabetes, and HF. In addition, obesity is associated with comorbidities like hypertension and sleep apnea, which further predispose individuals to AF. The treatment of AF in obese patients with OSA requires a multifaceted approach. Lifestyle changes, including weight loss, are critical in reducing both the burden of obesity and OSA, with studies showing that weight loss can reverse many of the structural and electrical changes contributing to AF.^32^ Bariatric surgery, for instance, has been shown to reduce the risk of AF recurrence after ablation, likely through reductions in epicardial adipose tissue volume, insulin resistance, and systemic inflammation.^33^

## Role of Hypoxia and Intrathoracic Pressure Change

OSA is associated with the continuous and repetitive collapse of the upper airway during sleep. This results in dynamic intrathoracic pressure changes in distinct intracardiac transmural pressure gradients with each inspiratory and expiratory effort. These differ in different stages of sleep and can have nightly variations. Intrathoracic pressure swings induce transient shortening in the atrial effective refractory period as well as intrathoracic pressure fluctuations (up to 60 mm Hg) during inspiration against an occluded upper airway, causing dynamic transmural pressure gradients and myocardial stretch,^34,35^ increasing the odds of AF.

Long-term intermittent hypoxia and hypercapnia also shorten the atrial effective refractory period. The change from hypercapnia to eucapnia leads to the return of the atrial effective refractory period to baseline, resulting in less risk of AF inducibility.^34,36^ Persistent deoxygenation-reoxygenation increases reactive oxygen species and possibly vascular inflammation, leading to cardiac remodeling.

In rats (age >4 weeks), intermittent airway obstruction produces connexin dysregulation and atrial fibrosis, associated with atrial conduction abnormalities and increased AF susceptibility/duration.^37^ Thus, there is a signal toward early substrate change in the OSA-exposed atrium beyond the acute changes in atrial depolarization.

Sleep phenotypic profiles associated with incident AF offer novel and clinically relevant insights into the relationship between sleep disorders and AF. The hypoxia subtype was associated with a 48% higher incidence of AF, followed by the apneas+arousals subtype (22% increase) and the short sleep+non-rapid eye movement (REM) subtype (11% increase) compared with the long sleep+REM subtype as the reference group.^38^ Although promising, these findings need further investigation and validation, mainly through prospective, interventional studies targeted to reduce the AHI, attenuate hypoxia, reduce arousal, and increase the quality and duration of sleep.^39^

Cortical arousals triggered by obstructive respiratory events are a defining feature of sleep-disordered breathing. Although traditionally considered markers of disrupted sleep and associated with adverse cardiovascular outcomes, arousals may also play a physiologically adaptive role. By terminating apneas, arousals help restore airflow and limit the duration of hypoxemia, CO_2_ retention, and vagal predominance. The Multi-Ethnic Study of Atherosclerosis (MESA) Sleep study revealed that a lower arousal index was independently associated with a higher prevalence of AF, suggesting that failure to mount an appropriate arousal response may allow more prolonged exposure to autonomic and respiratory stressors.^19^This finding challenges the assumption that arousals are uniformly deleterious and highlights a more complex interaction between sleep physiology and atrial arrhythmogenesis.

## Role of the Autonomic Nervous System—A Potential Target for Modulation

**Figure 3.**
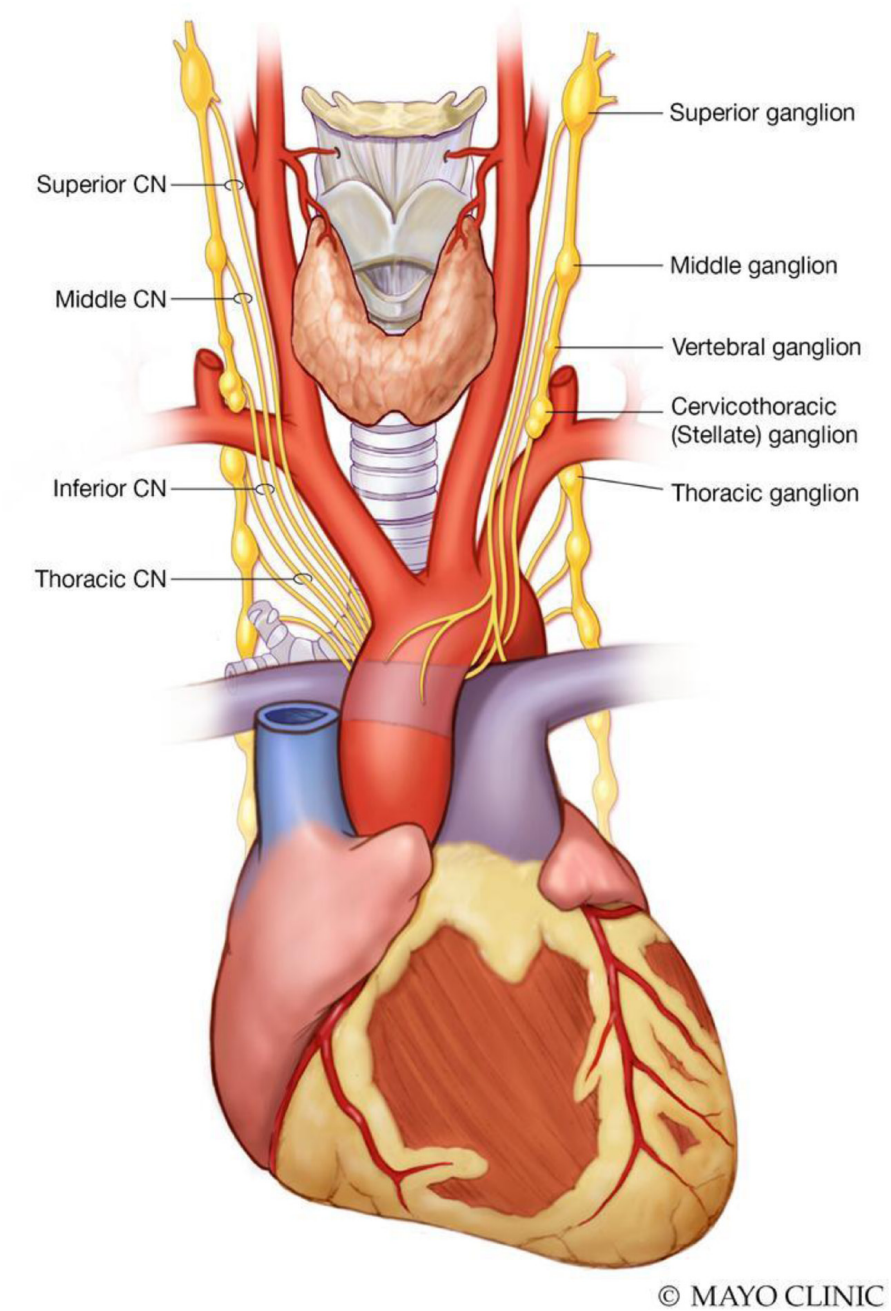
**Cardiac autonomic innervation.** CN indicates cranial nerve.

Growing evidence suggests that cardiac autonomic innervation is an essential link between OSA and AF. The cardiac autonomic nervous system comprises the external cardiac autonomic nervous system, central sympathetic and parasympathetic inputs, and the intrinsic cardiac autonomic system driven by the ganglionated plexi (GP) embedded in the various fat pads (Figure 3).

**Figure 4.**
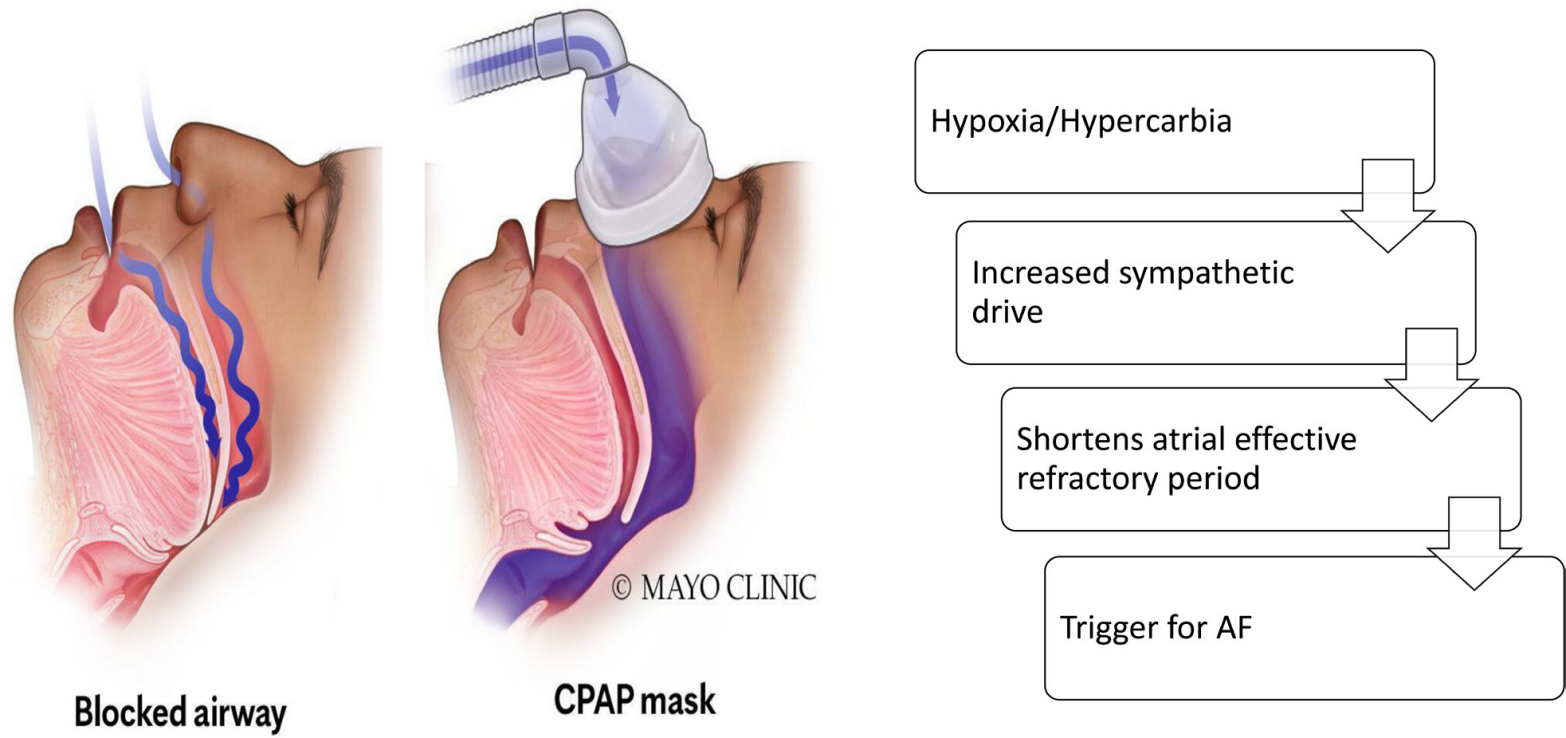
**Role of hypoxia in genesis for atrial fibrillation in patients with obstructive sleep apnea.** CPAP indicates continuous positive airway pressure.

Apnea-related hypoxia, respiratory acidosis, and hypercarbia can all be potential triggers for an increased sympathetic drive, which can result in hemodynamic and electrophysiological changes acting as a trigger for the initiation of AF (Figure 4). In an elegant study, Tavares et al,^40^ by inducing apnea in canine models, were able to document an increase in ganglionated plexus activity, increased phasic bursts of vagal activity resulting in elevated blood pressure (BP), heart rate, and increased sympathetic activity. These changes were noted with the shortening of the atrial effective refractory period and consistent induction of AF with a single atrial extra stimulus during apnea (easy AF inducibility). When GP sensory neurons were denervated with resiniferatoxin (the functional analog of capsaicin that activates transient vanilloid receptor 1), sympathetic and GP nerve activity decreased. Interestingly, AF inducibility during apnea did not occur due to the prolongation of the atrial effective refractory period.^40^ Ghias et al,^41^ who documented an increase in neural activity within the GP before AF initiation, corroborated these findings. AF induction was significantly mitigated after ablation of the GP in the vicinity of the right pulmonary artery. Future studies must investigate whether GP ablation could exert an antiarrhythmic effect by reversing chronic OSA-induced atrial remodeling.

In patients with OSA, increased muscle sympathetic tone nerve activity and skin sympathetic tone nerve activity have been reported. Simultaneous muscle sympathetic tone nerve activity and skin sympathetic tone nerve activity have been correlated with OSA,^42^ with arousals at the end of an apneic episode associated with large muscle sympathetic tone nerve activity and skin sympathetic tone nerve activity bursts. Thus, altering sympathetic tone, which is also elevated during an AF event, can be leveraged as a potential target to reduce the AF burden.

The role of the cardiac autonomic nervous system is also evident in affecting the electrophysiological properties of the atrial myocardium during obstructive apnea, suggesting cardiac afferents as a possible therapeutic target for autonomic modulation. Innovation in AF modulation to target autonomic dysregulation has formed the basis for interventional electrophysiological procedures.^43,44^ Contemporary neuromodulatory interventions have targeted the ganglia and nerves by catheter or surgical approaches. Beta-adrenergic blockers and other atrioventricular nodal agents may blunt the autonomic instability associated with obstructive respiratory events during sleep. By attenuating the sympathetic surges and abrupt heart rate accelerations that often follow arousals, these agents may help suppress postapneic ectopy and reduce the likelihood of arrhythmia initiation in patients with OSA.^45^

## Impact of OSA on Atrial Substrate and Triggers

OSA likely promotes AF via stretch-mediated shortening of the atrial refractory period and slow conduction mediated by collagen deposition that affects electrical propagation.^46^ The mechanical stretch of the atria by changes in intrathoracic pressures shortens the atrial refractory period. It promotes spontaneous atrial premature depolarizations, triggering episodes of AF in animal models of sleep apnea and humans.^47^

Anter et al^48^ examined the frequency and significance of triggers and abnormal substrate in patients with paroxysmal AF and OSA. They compared 43 patients with paroxysmal AF with moderate-to-severe OSA and without OSA who underwent pulmonary vein (PV) isolation (PVI) and ablation of nonpulmonary vein triggers. These 2 groups were compared with control groups of patients with and without OSA in whom only PVI was performed without ablating other nonpulmonary vein triggers. They noted a higher prevalence of low-voltage areas and abnormal electrograms both in the right atrium and left atrium in patients with OSA than without OSA. The left atrium, predominantly the left atrial septum, had more evidence of fibrosis based on the voltage map. As anticipated, the pulmonary vein triggers were common in both groups; however, after PVI, patients with OSA had an increased incidence of additional non-PV triggers (41.8% versus 11.6%; *P*=0.003). More recently, Nalliah et al^26^ reported increasing AHI correlated with an increasingly remodeled left atrium characterized by lower bipolar voltage, more significant voltage/conduction heterogeneity, and larger proportions of low-voltage areas. The effect of the severity of OSA on atrial remodeling appears to be dose-dependent, even in patients with paroxysmal AF. In contrast, persistent AF is associated with advanced atrial remodeling across categories of AHI. Thus, early and prudent OSA management in AF may have significant implications for identifying the right patient to benefit from PVI.

This was further clarified in an elegant study by the same group when they evaluated 24 patients with OSA (untreated) who underwent right atrial electrophysiological mapping, followed by randomization to either continuous positive airway pressure (CPAP) therapy—the recommended treatment for OSA—or no OSA therapy. After >6 months, these patients underwent a repeat invasive right atrial mapping study. They demonstrated that effective CPAP therapy resulted in faster atrial conduction velocity and improved atrial bipolar voltage than no CPAP therapy, independent of changes in systemic BP or weight. This study, although not powered for AF outcomes, clearly demonstrated the impact of OSA on atrial tissue characteristics and showed a positive change with CPAP therapy. It will be interesting to see changes in the left atrium with a similar strategy.

## Impact of OSA Treatment on Outcomes in Patients With AF

**Figure 5.**
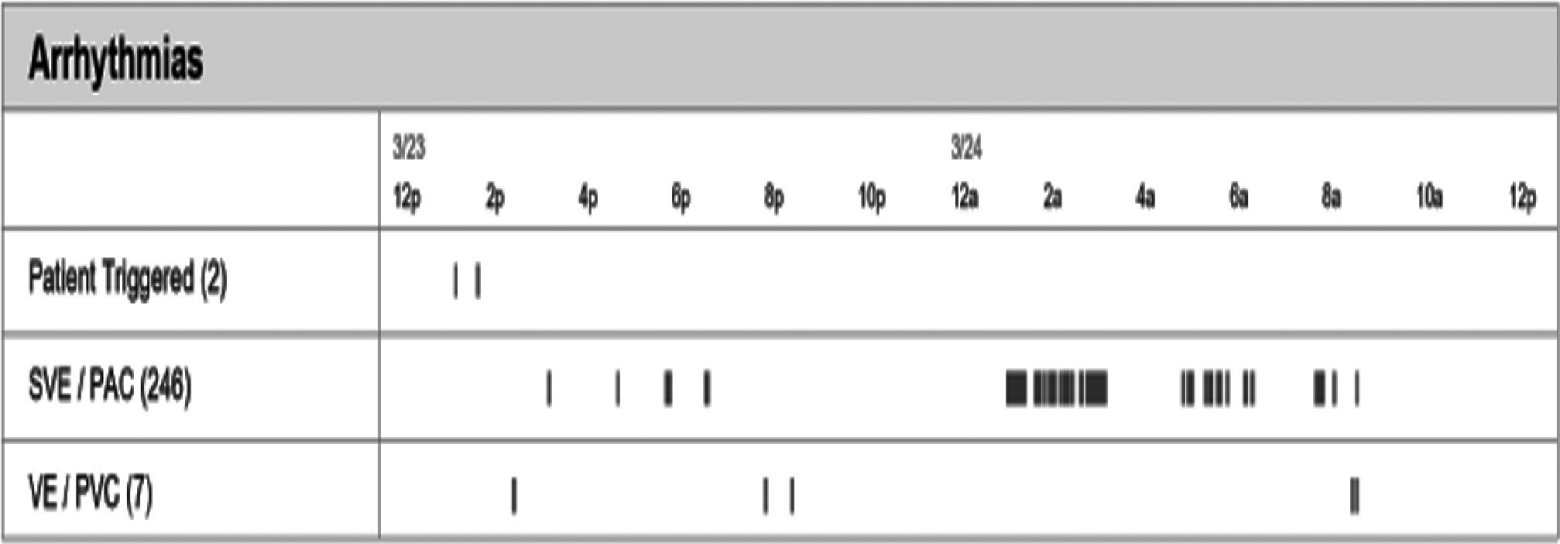
**Holter monitor of a patient showing enhanced atrial ectopy when not wearing a continuous positive airway pressure.** PAC indicates premature atrial contraction; PVC, premature ventricular contraction; SVE, supraventricular event; and VE, ventricular ectopy.

The robust association of OSA and AF would lead one to surmise that effective and early treatment of OSA can further improve rhythm control after AF ablation. Several observational studies have shown the importance of CPAP therapy in patients with OSA, as the patients not treated with CPAP are significantly more likely (2–6-fold) to have recurrent AF than CPAP-treated patients (Figure 5).^36,49,50^ A randomized clinical trial (25 out of 180 randomized patients) comparing CPAP to usual care for the prevention of recurrence after electrical cardioversion of AF showed no significant difference in outcome between the 2 groups.^51^ A recent larger randomized clinical trial using implanted loop recorders for AF detection tested the effect of 5 months of treatment with CPAP plus usual care versus usual care alone on AF burden (percentage of time in AF) in 109 patients with paroxysmal AF who were found on screening to have moderate or severe OSA. Despite reasonable compliance with CPAP (nightly use of 4.4 hours), the adjusted difference in AF burden between CPAP and usual care was 0.6% (*P*=0.52), and no significant difference was noted in the reduction in AF duration or burden.^52^

One of the most extensive retrospective reports among 3000 consecutive patients undergoing AF ablation noted that ≈21.3% had OSA. The patients with OSA had a higher risk of recurrence (*P*=0.024) and more nonpulmonary vein triggers (20% versus 8%, *P*<0.001). The patients with OSA also had more nonpulmonary vein triggers than those without OSA (31% versus 19%, *P*=0.001) in patients with nonparoxysmal AF. CPAP intervention significantly reduced the rate of AF recurrence (79% versus 68%, *P*=0.003).^53^ Similarly, in another retrospective report, among 426 patients undergoing PVI, 62 had documented OSA. CPAP therapy resulted in a higher AF-free survival rate (71.9% versus 36.7%; *P*=0.01) and AF-free survival off antiarrhythmic drugs or repeat ablation after PVI (65.6% versus 33.3%; *P*=0.02).^54^

In a meta-analysis of 1207 patients undergoing PVI, including randomized trials and observational studies, the participants were divided into a CPAP group (50.86%) and a non-CPAP group (49.14%). 33.52% had recurrent AF, and the recurrence rate differed between the CPAP and non-CPAP groups (24.88% versus 42.47%; *P*<0.001) after a mean follow-up of 16.33±10.34 months. Those patients treated with CPAP had a lower risk of recurrent AF after catheter ablation than those who did not, and about 17.59% of cases with recurrent AF could be attributed to not receiving CPAP. There was also a signal toward improvement in left atrium size and left ventricular ejection fraction (LVEF) post-ablation.^55^

The CABANA (Catheter Ablation vs Antiarrhythmic Drug Therapy for Atrial Fibrillation) trial showed no difference in the primary end point of the composite of death, disabling stroke, serious bleeding, or cardiac arrest in patients with and without OSA undergoing AF ablation versus antiarrhythmics (*P*<0.34).^56^ It is important to note that many randomized controlled trials (RCTs) evaluating the impact of PAP therapy on cardiovascular outcomes, including AF, often exclude symptomatic patients, especially those with severe daytime sleepiness, due to ethical concerns, potentially biasing results toward the null and limiting comparability with observational studies where more symptomatic patients are included.

Therefore, the key is to find those patients with OSA-AF in whom the OSA contributes to the genesis and maintenance of AF. Thus, future prospective trials to evaluate the impact of treating OSA in patients undergoing AF ablation will need a specific focus on identifying the subgroup of patients with OSA who are most likely to benefit from AF ablation.

## Central Sleep Apnea

Although the relationship between OSA and AF is well-established, the pathophysiological connection between CSA and AF remains less well-defined.

CSA is defined by reduced or absent respiratory effort during sleep, leading to recurring episodes of inadequate ventilation and impaired gas exchange. These disturbances result in oxygen desaturation and sometimes daytime sleepiness or insomnia. Unlike OSA, where hypoventilation arises from physical obstruction of the upper airway, CSA stems from a dysfunction in the brainstem, where signals to the respiratory muscles, including the diaphragm, are not transmitted effectively.^57^

Several studies have confirmed the increased incidence of AF in CSA patients.^58^ There is considerable overlap between the genesis of AF in patients with OSA and CSA. These include apnea-induced hypoxia with intermittent arousals, fluctuating CO_2_ levels, heightened sympathetic and neurohormonal activation, and oxidative stress leading to inflammation. However, there are some unique differences. Obstructive respiratory events are caused by mechanical obstruction of the upper airway during sleep. At the same time, central apneas result from impaired respiratory regulation at the brainstem, leading to cycles of hyperventilation and hypoventilation with fluctuating tidal volumes and carbon dioxide levels. These oscillations stem from a heightened sensitivity of the respiratory control centers to changes in PaCO_2_, commonly seen in patients with HF. The abrupt transitions between apnea and ventilatory overshoot provoke surges in sympathetic nervous system activity. This sympathetic overdrive is particularly pronounced during the hypercapnic and arousal phases and contributes to BP variability, heart rate turbulence, and increased myocardial oxygen demand. Over time, these neurohumoral shifts promote atrial remodeling via oxidative stress, inflammation, and electrical instability.

This heightened sympathetic tone, combined with intermittent hypoxia and mechanical stress, creates a vulnerable atrial substrate and increases the risk of AF.

The intrathoracic pressure swings accompanying OSA during ineffective inspiratory efforts against a closed airway are absent in CSA. CSA can present as 2 distinct phenotypes: (1) hypercapnic, characterized by elevated PaCO_2_ due to reduced respiratory drive from chemoreceptor insensitivity, which worsens with sleep onset, and (2) hypocapnic, with normal to low PaCO_2_, often secondary to conditions like HF or idiopathic CSA, driven by increased chemosensitivity.^59^ These fluctuations in PaCO_2_ and chemoreceptor sensitivity are associated with autonomic dysregulation and electrical remodeling, predisposing patients to AF.^60,61^

CSA has emerged as a significant predictor of AF in epidemiological studies. In the prospective Sleep Heart Health Study, CSA (defined by central apnea index ≥5 or Cheyne-Stokes breathing) was associated with a ≈2- to 3-fold higher odds of incident AF. Similarly, in the MrOS (Osteoporotic Fractures in Men) study, CSA and Cheyne-Stokes breathing predicted prevalent AF in older men. However, we acknowledge that such associations may reflect residual confounding—for example, CSA in these populations could be a marker of underlying HF or other comorbidities that themselves increase AF risk.

CPAP has traditionally been the first-line therapy for symptomatic patients with hyperventilation-related CSA. In the CANPAP trial (Canadian CPAP for Patients With CSA and HF), CPAP did not affect heart transplant-free survival. In a post hoc analysis, no statistically significant differences existed between the CPAP-CSA–suppressed/unsuppressed and control group in frequency of AF.^62^ Adaptive servo-ventilation is a form of positive airway pressure that remains a therapy option for patients with hyperventilation-related CSA and HF with preserved ejection fraction. A substudy from the CAT-HF (Catheter Ablation vs Standard Conventional Therapy in Patients with AF and HF) trial demonstrated proof of concept that adaptive servo-ventilation combined with optimized medical therapy led to a reduction in AF.^63^ However, large randomized controlled studies investigating the effect of CSA/Cheyne–Stokes Respiration (CSR) treatment on AF outcomes are not available. Phrenic nerve stimulation using the Remede system (Respicardia, Inc) is widely used to treat CSA. However, there are limited reports to attest to its benefit in AF.^64^ Thus, it is unsurprising that CSA is not addressed, and no specific screening/diagnostic or treatment recommendations are provided in the most recent AF guidelines.^65^

Importantly, AF is also prevalent, if not more so, in idiopathic CSA, in the absence of HF or other cardiac disease. Leung et al^66^ compared AF prevalence in 60 patients with idiopathic CSA (AHI>10 events per hour, >50% central events; all without a history of HF, coronary disease, or stroke) with 60 patients with OSA (AHI>10, >50% obstructive events) and 60 patients without sleep apnea (AHI<10), matched for age, sex, and BMI. Hypertension was most prevalent, and oxygen desaturation was most severe in the group with OSA. However, AF prevalence in idiopathic CSA was highest at 27%, compared with 1.7% in those with OSA and 3.3% in those without sleep apnea (*P*<0.001).^66^ These data indicate a strikingly high prevalence of AF in idiopathic CSA in the absence of OSA, hypertension, and severe deoxygenation, and argue for further studies of the mechanisms of AF in idiopathic CSA.

The interrelationship between sleep apnea, HF, and AF is complex and multifaceted, involving shared pathophysiological mechanisms and bidirectional influences. The interplay between sleep apnea, HF, and AF creates a vicious cycle where each condition can exacerbate the others. For instance, OSA can worsen HF by increasing sympathetic activity and promoting fluid retention, which in turn can lead to CSA. Both OSA and CSA can trigger AF through mechanisms such as atrial stretch, hypoxia-induced atrial remodeling, and autonomic imbalance.^67^ The presence of AF in patients with HF is associated with increased morbidity and mortality, making the management of both conditions crucial for improving patient outcomes. AF may worsen diastolic filling and impair cardiac output in patients with HF, creating a vicious cycle further amplified by untreated OSA. In addition, OSA-related surges in BP and heart rate variability during sleep can destabilize hemodynamics in patients with already compromised ventricular function. Several observational studies have demonstrated that untreated OSA is associated with increased rates of AF recurrence after cardioversion or catheter ablation. Although many of these studies were not exclusively limited to patients with HF, the pathophysiological mechanisms—atrial stretch, sympathetic activation, and hemodynamic instability—are particularly relevant in this population. In patients with concomitant HF, the presence of untreated OSA may further impair rhythm control, underscoring the importance of integrated sleep disorder management in AF-HF care pathways.^68^ Furthermore, in patients with HF especially, fluid retention and nocturnal rostral fluid shifts can worsen OSA due to upper airway edema and narrowing. Rostral fluid shifts with lung congestion, inducing hyperventilation and hypocapnia, may also contribute to CSA.^69^

## Beyond Sleep Apnea

Aside from sleep-disordered breathing (SDB), the contribution of other sleep disorders and dimensions to AF pathophysiology and prognosis is becoming increasingly apparent, as discussed below, and recognized by leading agencies.

## Insomnia

Insomnia is a common sleep disturbance, broadly defined as a perceived difficulty falling asleep, maintaining sleep throughout the night, or waking up too early in the morning, causing nonrestorative sleep. This, in turn, may cause daytime functional impairment, including fatigue, mood swings, and cognitive dysfunction. It is estimated that approximately one-third of adults report at least 1 insomnia symptom and that 6% to 10% of the general population meet the diagnostic criteria for insomnia disorder.^70^ The burden of insomnia is partially owed to the excess morbidity and mortality risk portended by this sleep disorder, including heightened odds of CVD. Accordingly, insomnia is now recognized as a powerful risk factor for hypertension, coronary heart disease, HF, stroke, and cardiovascular death.^71–73^ Of note, the adverse consequences of insomnia are potentiated when sleep difficulties are accompanied by short sleep duration.^74^ However, other studies show that insomnia severity and symptoms predict CVD independently of sleep duration.^75–77^

Convincing evidence linking insomnia to cardiac arrhythmias and AF is emerging. Poor sleep is a frequent complaint in patients with AF. A secondary analysis of the I-STOP-AFIB (Individualized Studies of Triggers of Paroxysmal Atrial Fibrillation) trial revealed that worse perceived sleep predicted a 15% increased risk of reporting AF episodes the following day, with a commensurate relationship noted between sleep quality categories and duration of reported AF events.^78^ Several cross-sectional studies describe a higher prevalence of AF in those with insomnia than in good sleepers, independently of known confounders.^79–82^ A community-based investigation found that people with insomnia, as determined by a questionnaire, were 1.92× (95% CI, 1.00–3.70) more likely to suffer from AF than those without insomnia.^79^ This association was more conspicuous in those aged <40 years, suggesting greater susceptibility in younger adults. However, in other studies, a significant relationship between insomnia and AF was also observed in the elderly.^80,81^

Insomnia has been found to predict future AF in multiple longitudinal examinations.^83–88^ In a cohort of over 1 million US veterans followed up for an average of 10 years, the risk of developing new-onset AF was 32% higher in those with a diagnosis of insomnia than in controls after accounting for demographic, lifestyle, and clinical covariates, including OSA.^84^ Assessment of the relation between insomnia and AF withstood adjustment for OSA in other studies,^85,86^ giving strength to the notion that, despite OSA and insomnia often co-occurring,^89^ the latter sleep disorder conveys unique, independent implications for AF. Relatedly, it is notable that insomnia’s predictive power for AF may be comparable to or even exceed that of OSA.^83,87^ Multivariable-adjusted data from a nationwide Taiwanese population cohort show that insomnia at baseline yielded a 1.26-fold more significant risk of developing new-onset AF, while the corresponding hazard ratio of OSA was 1.19.^87^ Similar considerations can be extended to depression, a well-documented precursor of AF,^90^ and frequently comorbid with insomnia,^91^ with several investigations revealing similar or greater estimates of AF risk conferred by insomnia in comparison with depression.^83,85,86,88^ These observations further reaffirm the relevance of insomnia for AF risk beyond conventional precursors.

In line with prevalence data, the association between insomnia and incident AF may be modified by demographic characteristics, with a more pronounced impact of poor sleep noted in younger and middle-aged adults.^86,87^ Although findings of Lee et al^83^ indicate heightened vulnerability to the arrhythmogenic effect of poor sleep in males, no sex differences emerged in other studies.^85–87^

The prognostic significance of insomnia also manifests in relation to AF treatment. In a sample of Chinese patients receiving ablation for AF,^92^ rates of long-term postablation AF recurrence were more elevated in patients reporting insomnia than in those without it. Furthermore, a dose-dependent relationship was noted—the higher the number of insomnia symptoms, the greater the hazard of AF relapse, with patients reporting ≥3 symptoms exhibiting more than twice the risk. Insomnia may also contribute to worsening AF prognosis by affecting adherence to therapeutic regimens. Patients with AF who complained of poor sleep compromising daily functioning also exhibited lower compliance with pharmacotherapy, with the likelihood of medication nonadherence rising with increasing frequency of daytime impairment.^93^

Although the pathophysiological underpinnings of AF-related insomnia remain elusive, various mechanisms are thought to be implicated. Grounded on the concept that insomnia can be regarded as a state of physiological and psychological hyperarousal,^94^ sympathetic overdrive has been proposed as the chief mechanism underlying the proarrhythmogenic effects of insomnia. Findings of elevated plasma norepinephrine,^95^ suppressed heart rate variability,^96,97^ along with accelerated heart rate,^96,98–100^ corroborate this hypothesis. Exaggerated muscle sympathetic nerve activity and BP reactivity to stress in insomniacs^101^ further support altered cardiovascular autonomic control in this condition. Although speculative, activation of the renin-angiotensin-aldosterone system, potentially via sympathetic stimulation, could contribute to insomnia-induced BP dysregulation.^102^ This would promote electrical and structural remodeling of the atrium and atrial fibrosis, thus favoring an arrhythmogenic milieu. Upregulation of the hypothalamic-pituitary-adrenal axis, consistent with data on increased cortisol levels in insomniacs,^103–105^ may also be implicated in the excess risk of AF conferred by insomnia. Other potential pathways may involve inflammation, with supporting evidence derived from studies documenting an association between insomnia and elevation in inflammatory cytokines such as CRP (C-reactive protein) and IL (interleukin)-6.^106,107^ However, because negative findings have also been reported,^108,109^ it remains unclear whether insomnia elicits a proinflammatory state that could play a role in AF development. It is plausible that these mechanisms, especially hyperarousal-related perturbations, may be linked to abnormal sleep architecture, quantity, and continuity exhibited by insomniacs, as evidenced by polysomnography studies.^110^ The concept of sleep instability, and primarily REM sleep instability, has recently been implicated in insomnia and underlying hyperarousal.^111^ Short sleep duration is also thought to play a key role in the pathophysiology of insomnia and its complications, as it activates multiple CVD pathways, including those discussed above. An overview of these mechanisms is provided later in this review.

Last, support for a causal relationship between insomnia and AF arises from Mendelian randomization studies.^112,113^ Leveraging genome-wide association studies data from large consortia and the UK Biobank, Liu et al^112^ found that multivariable-adjusted odds for AF were 1.13-fold higher (95% CI, 1.08–1.18) in individuals with a genetic predisposition to insomnia. Of note, BMI and BP partially mediated the relationship between genetic liability for insomnia and AF.

Cognitive behavioral therapy for insomnia (CBT-I) is the recommended therapy for this sleep disorder.^114,115^ CBT-I is a multicomponent regimen that integrates cognitive strategies, behavioral skills, and sleep education to target predisposing and perpetuating factors causing insomnia. CBT-I is preferred to drug therapy due to its superior long-term efficacy and absence of complications. Intriguing clinical trial findings suggest that alleviating insomnia through CBT-I may also enhance cardiovascular health, with this intervention’s positive biological impact on multiple cardiometabolic risk indicators.^116^ Although evidence of the effects of CBT-I for insomnia and related outcomes in patients with AF is lacking, a randomized controlled trial of 10 weeks of CBT versus education for symptom preoccupation in AF also showed ameliorated symptoms of insomnia severity after the intervention.^117^ Furthermore, evidence indicates partial insomnia remission following AF treatment. In patients with AF who underwent cardioversion, sleep quality, as assessed by the Pittsburgh Sleep Quality Index, improved in patients who maintained sinus rhythm at 6 months.^82^ Similarly, a marginal improvement in subjective sleep was recorded after 3 months post-ablation, with greater benefits in men and irrespective of AF severity.^118^ Given the paucity of data on the impact of insomnia therapy on both sleep health and AF outcomes, well-designed clinical trials yielding robust evidence are warranted.

## Narcolepsy

Narcolepsy is a chronic sleep-wake disorder characterized by excessive daytime sleepiness, typically presenting with cataplexy, hypnagogic/hypnopompic hallucinations, sleep paralysis, and disrupted nighttime sleep. Laboratory findings include ≥2 sleep-onset REM episodes and objective sleep latency ≤8 min on the multiple sleep latency test.^119^ The presence of cataplexy and undetectable levels of hypocretin in the cerebrospinal fluid (<110 pg/mL) distinguishes narcolepsy type 1 from narcolepsy type 2.^120^ The cause of narcolepsy is multifactorial and includes genetic and environmental determinants. Loss of hypocretin-producing neurons, possibly of autoimmune origin, is the core disease mechanism of narcolepsy type 1, whereas the pathophysiology of narcolepsy type 2 is poorly understood.^121^ It is estimated that about 25 to 50 cases of narcolepsy occur per 100 000 individuals, with a higher prevalence of narcolepsy type 2.^122,123^

Narcolepsy is a profoundly debilitating disease due to its disabling symptoms affecting daily functioning and quality of life. The burden of narcolepsy is exacerbated by the high medical and psychological multimorbidity suffered by these patients.^124^ As reviewed elsewhere,^125^ overt CVDs and cardiovascular risk factors such as hypertension, nondipping BP profiles, dyslipidemia, and obesity are prevalent in patients with narcolepsy. As it pertains to AF, a large study of administrative claims found that crude incidence rates of AF were higher in patients with narcolepsy than in matched controls (5% versus 3.2%).^126^ Nevertheless, the association was not retained following multivariable adjustment. Similarly, an east Europe–based epidemiological study of narcolepsy reported a comparable prevalence of cardiac arrhythmia/AF between cases and controls.^127^ Although these observations provide little support for an independent association between narcolepsy and AF, definitive conclusions cannot be drawn due to the current paucity of data, and further investigations on this subject are needed.

Because the therapeutic regimen for narcolepsy typically includes stimulants and wake-promoting agents, concerns for potential iatrogenic effects on the cardiovascular system, including diurnal and nocturnal hypertension and AF risks, have also been raised.^128^ Sodium oxybates, another common therapy for narcolepsy, contain elevated quantities of sodium, and excessive dietary sodium can predispose to AF development.^129,130^ It is noteworthy that novel low-sodium formulations have recently been made available, conceivably diminishing cardiovascular consequences. Relatedly, recommendations to mitigate cardiovascular risk in narcolepsy patients include reducing sodium intake.^131^

## Inadequate Sleep Duration

Expert panels’ evidence-based recommendations advise that adequate sleep duration for adults ranges between 7 and 9 hours per night, whereas 7 to 8 hours may be appropriate for older adults.^132,133^ These guidelines are predicated on a compelling body of research showing that departures from this amount of sleep, in either direction, are associated with a heightened risk of a multitude of medical and psychiatric disorders and reduced survival.^134,135^ However, a substantial proportion of the population reports habitual sleep duration outside the recommended ranges, with short sleep being especially prevalent. Contemporary data from >400 000 US adults surveyed by the Behavioral Risk Factor Surveillance System show that 36.8% of the respondents report sleeping <7 hours/night 135, and population-based studies suggest a high prevalence of short sleep duration globally.^136–139^ Accordingly, the epidemiological burden of insufficient sleep and its public health ramifications are increasingly recognized. Robust observational and experimental evidence identifies short sleep duration as an independent determinant of subclinical and clinical manifestations of CVD and a predictor of premature cardiovascular death.^140–142^

Data linking chronic sleep deficiency to augmented AF risk are also accumulating. In patients with hypertension, odds of prevalent AF associated with sleep duration of ≤ 5 hours/night compared with 6 to 8 hours were 1.95 (95% CI, 1.28–2.95) in fully adjusted analysis accounting for established mediators of risk, while no association was noted in those reporting ≥9 hours of sleep.^143^ A prospective evaluation of a Japanese cohort of 6898 individuals aged 30 to 84 years found that sleeping ≤6 hours was independently associated with a 34% greater risk of incident AF.^144^ Short sleep precipitates AF recurrence after ablation, and each hour, a 1-hour increase in sleep duration lowers the risk of recurrent AF by 14%.^92^ Findings from studies that objectively quantified sleep integrate evidence based on subjective, self-reported sleep duration. In multivariable-adjusted analysis, risks of prevalent and incident AF increased by 1.17 (95% CI, 1.11–1.30) and 1.09 (1.05–1.13) times, respectively, per each reduction in sleep time as measured from polysomnography.^145^ Consistently, a longitudinal examination of data from the UK Biobank found that accelerometer-derived sleep duration <7 h/d predicted future AF over a 7-year follow-up period.^146^

In contrast with these observations supporting a predictive role of reduced sleep only, other studies suggest a U-shaped association between sleep duration and AF, with both extremes of sleep time portending a higher risk of AF compared with normal sleep.^147^ Moreover, a large population-based cohort study found that those sleeping ≥8 hours were more likely to develop AF than those sleeping 7 hours, whereas sleeping ≤6 hours was not predictive.^148^ Long but not short sleep duration prognosticated higher risk of AF recurrence after ablation^149^ and higher AF/atrial flutter mortality.^150^ These findings would indicate that both ends of the sleep duration continuum may be adversely related to AF. Nevertheless, results of Mendelian randomization studies investigating the link between genetic variants associated with short and long sleep durations and the risk of developing AF converge to identify only short sleep as a significant potential causal risk factor for AF.^151,152^ Using a large genome-wide association study to identify single-nucleotide polymorphisms associated with sleep duration, Ai et al^151^ found that the odds of AF decreased by 32% per each 1-hour increase in sleep duration. Because Mendelian randomization studies can yield unbiased estimates, obviating caveats of observational investigations, these findings suggest that the implications of long sleep for AF risk, as found in some cohorts, may be distorted by unmeasured risk factors, residual confounding, or reversed causality. The lack of experimental evidence on the adverse effects of prolonged sleep on cardiovascular health, paralleled by compelling data on the detrimental impact of short sleep, further corroborates this notion, as discussed below.

**Figure 6.**
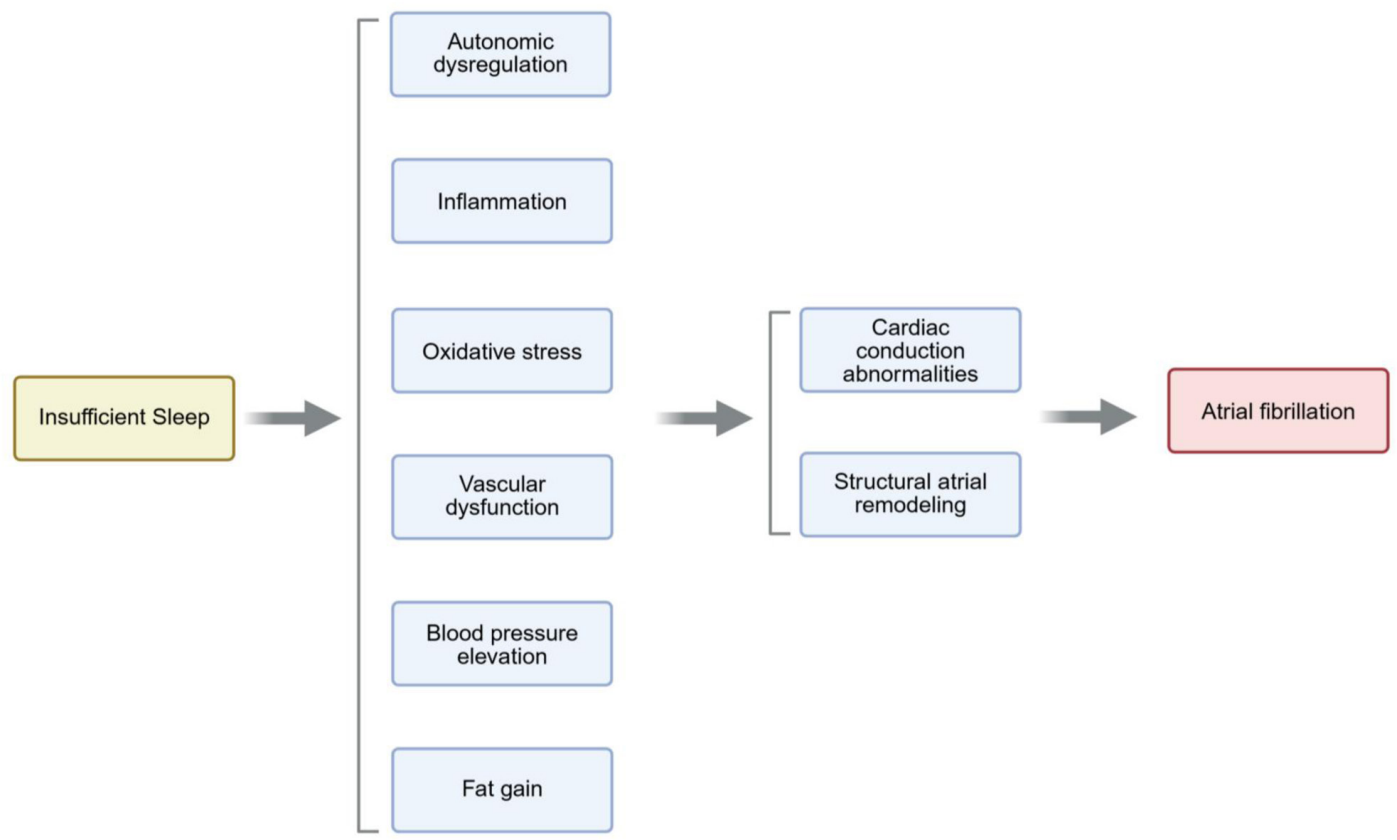
**Putative mechanisms linking insufficient sleep to heightened risk of atrial fibrillation.** Created in BioRender.

Laboratory-based studies of experimentally induced short sleep also shed light on biological mechanisms that may mediate the arrhythmogenic impact of short sleep (Figure 6). Healthy young adults exposed to one night of sleep deprivation exhibit P-wave prolongation and increased P-wave dispersion the following morning.^153^ Autonomic perturbations caused by sleep loss likely drive such abnormalities, as suggested by findings of increased urinary and plasma catecholamines,^154–156^ and altered heart rate variability (HRV) and baroreflex during experimental sleep restriction.^155,157,158^ Although data on changes in heart rate are mixed, sympathetic hyperactivity is also thought to contribute to BP increases exhibited in response to total and partial sleep deprivation,^154,157,159,160^ which, in the long term, would promote conduction defects and structural alterations in the atrium and favor AF inducibility. Increased systemic and tissue inflammation and oxidative stress in sleep-deprived study participants^161–163^ may mediate the endothelial function impairment produced by sleep loss.^154,155,164^ This would trigger atrial remodeling, thereby favoring AF substrate development. In addition, short sleep causes increased energy intake, possibly by altering the neuroendocrine control of appetite, thus promoting weight gain and obesity. This may also contribute to the excess risk of AF portended by insufficient sleep.^165–167^

Restoration of normal sleep duration may reverse the unfavorable risk profile of short sleepers. Studies applying sleep extension interventions provide attractive evidence to support this idea. Improved markers of cardiovascular health, including decreased BP and weight loss,^168,169^ have been reported in response to behavioral sleep extension programs. While plausible, it is unknown whether sleep duration normalization would reduce susceptibility to AF and associated complications, and thus awaits investigation.

## RLS and Periodic Leg Movements During Sleep

RLS is a common sensorimotor disorder characterized by an urge to move. It occurs predominantly during periods of rest, in the evening, and at night, and improves with movement.^170^ Patients with RLS also have insomnia and involuntary periodic leg movements (PLM) during sleep (PLMS), which are often associated with autonomic activation, increased nocturnal BP, hypertension, and CVD, including AF. Although the role of OSA in AF risk has been widely studied, the impact of RLS and PLMS on AF remains unclear. However, several cross-sectional and longitudinal studies reported a potential association between RLS, PLM, and AF.

One study found that among patients with RLS, those with high PLMS (>35/h) were older, predominantly male, with lower BMI, and had increased prevalences of coronary artery disease and AF.^170^ The same group reported that AF was 8% prevalent among a larger population of 4951 consecutive patients with a clinical suspicion of RLS.^171^ Follow-up data were available in a subpopulation of 373 patients with nonpermanent AF, 77% with paroxysmal and 23% with persistent AF. Patients with PLMS >35/h tended to have more persistent AF than those with lower PLMS.^171^ The longitudinal component of the study showed that patients with frequent PLMS had an increased risk of having AF progression (ie, arrhythmia persistence, need for additional arrhythmia therapy, failure of electrical cardioversion, or switching from nonpermanent to permanent AF) than those with lower PLMS. Multivariate analysis showed that PLMS >35/h was an independent predictor of AF progression. The effect of treatment of RLS and PLMS with dopaminergic drugs was assessed in a subgroup of 153 patients, but no difference was found in the progression of AF between treated and untreated patients in the group with a low PLMS index. In contrast, AF progression was 2.7-fold lower in the treated group with high PLM compared with the untreated group with high PLM.^171^

Another study, using a claims database and data collected retrospectively but with subjects followed prospectively for 8 years, assessed whether participants with RLS but free of CVD at baseline had increased incident risk of CVD (ie, myocardial infarction, angina, stroke, HF, AF) as a function of treatment for RLS (ie, dopaminergic, anticonvulsants, benzodiazepines, and opiates).^172^ The hazard ratio for the future development of AF was 1.17 (1.03–1.12) in patients with treated RLS and 1.88 (1.25–2.25) in nontreated RLS, each group compared with non-RLS patients. These important data suggested that RLS was associated with an increased risk of AF and that treating RLS may decrease the risk of AF.

Apart from RLS, several other studies have assessed the association between PLMS and AF in the general population and patients with SDB. In a cross-sectional study including 2793 community-dwelling older men, PLMS and those associated with arousals were not associated with AF after considering potential confounders. However, this association tends to be observed in participants with chronic HF or myocardial infarction.^173^ Using the same cohort, another study reexamined this association in elderly men without prevalent AF at baseline, prospectively with an 8-year follow-up. In the overall cohort, no association was found between PLM and PLM with arousals and AF; however, in men aged ≥76 years, participants with PLMS> 30/h had an increased risk of AF compared with those without PLMS (ie, <5/h).^174^ In a large population of 15 414 patients tested for sleep disorders at the Mayo Clinic, 76.3% had SDB, 13.1% had PLMS between 15 and 30/h, and 36.1% had PLMS above 30/h.^175^ Patients with PLM≥30/h and PLMS with arousal ≥5/h had a 2 and 1.7-fold increased risk of having AF, respectively, and a 2-, 2.5-, and 3-fold increased risk for mild, moderate, and severe SDB, respectively. However, after multiple adjustments, no association persisted with PLMS or PLMS with arousal. In contrast, in patients with mild SDB, those with PLM≥30/h and PLMS with arousal ≥5/h had a 1.2 and 1.3 increased risk of having AF, respectively.

Altogether, these findings suggest potential associations between RLS, PLMS, and susceptibility to cardiac arrhythmia and AF progression. The precise mechanisms underlying these associations remain unknown but may involve sleep fragmentation, acute BP increases, increased nocturnal sympathetic activation, inflammation, and iron deficiency.^170^ Additional studies are needed to better understand these associations, establish a potential causal relationship, and evaluate the impact of management in well-designed clinical trials. These studies could have important implications for screening and treating RLS as part of primary and secondary prevention of AF and AF recurrence, respectively.

## Other Sleep Characteristics and Habits

The implications of quantitative sleep architecture features and sleep habits for AF have been gaining increasing attention. Xie et al^176^ conducted a comprehensive assessment to evaluate subjective and objective sleep characteristics associated with AF in independent cohorts. Self-reported frequent nocturnal awakening was a significant predictor of prevalent and incident AF in the Health eHeart Study and the Cardiovascular Health Study, respectively. Of note, these associations were independent of conventional covariates and other sleep features, including OSA. Perceived long sleep latency was also independently associated with prevalent AF, whereas shorter REM sleep duration, as quantified from polysomnography, predicted a higher risk of new-onset AF. Conversely, a significant relationship between slow-wave sleep and AF was found in the Multi-Ethnic Study of Atherosclerosis Sleep study,^19^ with greater amounts of slow-wave sleep associated with reduced AF prevalence in maximally adjusted models, including AHI. These findings have been replicated in a large study using wearable data from the All of Us Research Program,^177^ showing that AF risk decreased with increasing duration of both REM and deep (slow-wave) sleep.

Daytime sleep habits have also been linked to AF. Those who reported taking naps during the day were 28% more likely to suffer from AF than non-nappers.^143^ Moreover, joint effects of short sleep and napping were noted, with individuals reporting sleeping ≤5 hours per night and napping being more than twice as likely to have AF than those sleeping 6 to 8 hours and not napping. Genetic predisposition to daytime naps has also been causally related to AF in Mendelian randomization studies. By assessing 91 single-nucleotide polymorphisms known to be associated with daytime napping, Zhang et al 170 found that genetically predicted napping predicted a 27% increased risk of future AF.^178^

More recently, evaluating multidimensional sleep patterns has unveiled novel implications for disease risk, including AF. Harnessing UK Biobank data, Li et at^179^ computed a sleep health score combining self-reported insomnia, snoring, sleep duration, daytime sleepiness, and chronotype information. A healthy sleep score, defined as a sleep duration of 7 to 8 hours, no excessive daytime sleepiness, no snoring or insomnia symptoms, and early chronotype, was associated with a 29% lower risk of developing AF compared with those with worse sleep scores, namely poor sleep patterns. Of note, this association was especially robust in individuals with a low genetic risk of AF, suggesting interaction with genetic predisposition. Poor composite sleep patterns also predicted transition from AF to cardiovascular multimorbidity,^180^ and postablation AF recurrence,^181^ with patients reporting unhealthy sleep manifesting more than a 3-fold greater risk of AF relapse than healthy sleepers.

Taken together, these observations indicate that adherence to healthy sleep behaviors beyond individual sleep disturbances is critical to mitigate the risk of AF and related outcomes.

## Screening for Sleep Disorders

With a renewed focus on risk factor modification for controlling AF, screening, diagnosis, and treatment of OSA have become paramount to improving patients’ outcomes.^182^

The European Society of Cardiology guidelines for diagnosing and managing AF recommend that screening for AF should be considered in patients with OSA.^65,183^ The American Academy of Sleep Medicine considers those with AF at high risk for SDB and recommends evaluation for OSA.^184^ The gold standard for diagnosing sleep apnea is overnight polysomnography, typically conducted in a sleep laboratory, which can be costly and inconvenient for patients.

The STOP Questionnaire (Snoring, Tiredness, Observed Apnea, High Blood Pressure), STOP-Bang Questionnaire (STOP Questionnaire plus BMI, Age, Neck Circumference, and Gender), Berlin Questionnaire, and the Multivariable Apnea Prediction tool are some of the screening tools. However, none of these have been adequately validated in a primary care setting, older patients, and even less so in patients with AF.^185^

Home sleep apnea testing offers a more accessible alternative, though these tools have not been extensively validated in patients with AF.

The MOODS-AF (Monitoring, Objective Outcomes and Definitions in Symptomatic Atrial Fibrillation) model was developed to address this gap. By incorporating clinical, echocardiographic, and rhythm-specific variables, it demonstrated improved discrimination compared with general population tools.^186^ Additional approaches incorporating physiological markers, such as heart rate variability or AF subtype, may further improve detection in asymptomatic individuals. May et al^187^ evaluated traditional screening instruments and proposed novel models specifically designed for the AF population, incorporating features such as nocturnal heart rate variability and AF phenotype. 

Finally, although OSA has received the most attention, other forms of sleep-disordered breathing—including CSA, complex sleep apnea, and disorders characterized by sleep fragmentation—also warrant consideration. A broader approach to sleep assessment in AF should incorporate evaluation for insomnia, periodic limb movements, and disrupted sleep architecture, especially in patients with recurrent arrhythmia despite treatment.

Therefore, a comprehensive approach to sleep assessment in patients with AF should include evaluation for multiple types of sleep disturbances, not solely OSA. Incorporating validated insomnia screening tools (eg, Insomnia Severity Index), assessing sleep architecture during polysomnography, and recognizing the cardiovascular implications of nocturnal arousals and nonrespiratory sleep fragmentation may help identify at-risk individuals. Future screening protocols and clinical pathways may benefit from a more holistic evaluation of sleep health to optimize rhythm control strategies in AF.^188^ Advances in digital health and wearable technologies may further enhance the ability to detect physiological signals suggestive of sleep disorders in ambulatory settings.

## Future Direction and Unmet Needs

As a modifiable risk factor, the impact of poor sleep on CVDs, including AF, is now acknowledged by leading organizations. The National Heart, Lung, and Blood Institute identified understanding sleep disturbances and disorders as a key scientific priority to advance strategies for AF prevention.^189^ In 2022, the American Heart Association recognized sleep as a critical component of cardiovascular health, expanding its former Life’s Simple 7 to include healthy sleep in the updated Life’s Essential 8.^190^ In parallel, contemporary AF management guidelines from the American College of Cardiology/American Heart Association/Heart Rhythm Society and the European Society of Cardiology now recommend routine screening for OSA in patients with AF.^65,182^ These aligned recommendations reflect the accumulating evidence that sleep-disordered breathing contributes to AF pathogenesis and may influence therapeutic response, reinforcing the need to incorporate sleep assessment into AF care pathways.

The number of patients diagnosed with AF and OSA continues to increase. With the significant burden on the healthcare system, a more innovative way to identify patients with OSA-AF and OSA, either with a better questionnaire design or advanced data or imagery analytics, will be the way forward.

A structural change in the atria is noted even in paroxysmal AF; whether early treatment of OSA can reduce the progression of paroxysmal AF to persistent or permanent AF is unknown. Similarly, whether regression of substrate abnormalities can be seen with treatment with CPAP awaits further study.

Several retrospective and observational studies have shown a clear signal toward improvement in AF outcomes and an overall reduction in burden in patients treated with OSA. However, it remains unclear why the same has not been seen in prospective randomized control trials, especially targeting AF by ablation. Certainly, PVI does not target the autonomic nervous system consistently; hence, whether any autonomic modulation with conventional ablation strategies may reduce the AF burden remains to be seen. Instead of looking at AHI, identifying whether other factors, such as sleep fragmentation, O_2_ desaturation, sleep quality and duration, and measures of hypoxic burden, may be crucial for the initiation and maintenance of AF may offer targets to mitigate them. The role of autonomic interplay is a promising landscape for targeting them invasively during ablation or modulation with wearables.

An important consideration in understanding the effects of OSA treatment in patients with AF and OSA is that OSA is common, and AF is common. Therefore, they often coexist. AF is also multifactorial. Because they may be comorbidities, it does not necessarily mean that the OSA is causing the AF. Thus, treating OSA in a patient with AF may not necessarily impact AF burden or recurrence. A pivotal aspect of future studies is to identify the subgroup of AF-OSA comorbid patients in whom OSA is likely contributing to the AF and, thus, where treatment of OSA may be reasonably expected to attenuate AF. Finally, whether effective control of OSA can reduce long-term sequelae of AF, such as strokes and dementia, warrants long-term investigations.

Further efforts should also be undertaken to better characterize susceptibilities to the arrhythmogenic impact of OSA and other sleep disturbances. The moderating role of demographic and clinical characteristics, as well as the impact of social determinants of health, on the excess risk of AF portended by poor sleep has not been adequately addressed, yet it would likely benefit risk stratification.

Importantly, there is overall a dearth of studies directly investigating mechanisms mediating the increased risk of AF in patients with insomnia, short sleep duration, sleep fragmentation, narcolepsy, or RLS. The proposed causal pathways are largely based on evidence of the implications of these sleep disturbances for other CVDs; little is known about their direct effects on the atrial substrate and triggers. More research is therefore needed to elucidate the pathophysiology of AF in those with insomnia, narcolepsy, or RLS as a function of REM versus nonrapid eye movement (NREM) sleep fragmentation.

Similarly, there is a paucity of evidence on antiarrhythmic effects of therapies for sleep disorders other than SDB. Although a variable degree of cardioprotection is inferred largely based on improvements in intermediate disease mechanisms, the impact of behavioral or pharmacological treatments for insomnia, RLS, and narcolepsy on AF incidence and progression is largely unknown. There is thus an urgent need for robust clinical trials targeting AF outcomes in patients with these sleep disorders. Furthermore, beyond clinical sleep diagnoses, subclinical manifestations of disrupted sleep have also been independently associated with a heightened risk of AF, as discussed above. Whether interventions aimed at ameliorating sleep practices, extending sleep duration, and improving sleep quality may confer benefits against AF awaits investigation.

## ARTICLE INFORMATION

### Acknowledgments

Figures were created by Mayo Clinic Media Support Services.

### Sources of Funding

V.K. Somers is supported by National Institutes of Health (NIH) HL65176 and HL134885. N. Covassin is supported by NIH HL169320.

### Disclosures

A. Deshmukh consulted for GE Healthcare; V.K. Somers serves on the Sleep Number Scientific Advisory Board and as a consultant for Lilly, Jazz Pharmaceuticals, ApniMed, iRhythm, Mineralys, and Axsome. Y. Dauvilliers received funds for seminars, board engagements, and travel to conferences from Avadel, Bioprojet, Idorsia, Jazz Pharmaceuticals, Centessa, and Takeda. The other author reports no conflicts.
